# Current understanding of the administration of mesenchymal stem cells in acute kidney injury to chronic kidney disease transition: a review with a focus on preclinical models

**DOI:** 10.1186/s13287-019-1507-3

**Published:** 2019-12-16

**Authors:** Lingfei Zhao, Fei Han, Junni Wang, Jianghua Chen

**Affiliations:** 1 Kidney Disease Center, the First Affiliated Hospital, College of Medicine, Zhejiang University, Hangzhou, Zhejiang Province People’s Republic of China; 2 Key Laboratory of Kidney Disease Prevention and Control Technology, Zhejiang Province, Institute of Nephrology, Zhejiang University, Hangzhou, Zhejiang Province People’s Republic of China

**Keywords:** Mesenchymal stem cells, AKI-CKD transition

## Abstract

Incomplete recovery from acute kidney injury (AKI) can result in long-term functional deficits and has been recognized as a major contributor to chronic kidney disease (CKD), which is termed the AKI-CKD transition. Currently, an effective intervention for this disorder is still lacking. Principally, therapeutic strategies targeting the AKI-CKD transition can be divided into those reducing the severity of AKI or promoting the regenerative process towards beneficially adaptive repair pathways. Considering the fact that mesenchymal stem cells (MSCs) have the potential to address both aspects, therapeutic regimens based on MSCs have a promising future. In light of this information, we focus on the currently available evidence associated with MSC therapy involved in the treatment of the AKI-CKD transition and the underlying mechanisms. All of these discussions will contribute to the establishment of a reliable therapeutic strategy for patients with this problem, who can be easily ignored by physicians, and will lead to a better clinical outcome for them.

## Background

Despite advances in modern medicine, the incidence, prevalence, and mortality of chronic kidney disease (CKD) have increased in the last 10 years [1]. CKD is characterized as a continuously advancing loss of renal function and irreversible accumulation of extracellular matrix. According to a US renal data system annual report, approximately 13.6% of people in the USA are suffering from CKD [2]. The high morbidity and poor prognosis place a huge burden on public health systems, and it is estimated that over 49 billion dollars are spent annually for treating CKD patients [3].

One major reason for the development of CKD is acute kidney injury (AKI). Contrary to the traditional view that AKI and CKD are considered as two distinct syndromes, numerous animal studies and epidemiological evidence in recent years provide a strong possibility that AKI and CKD share an interconnected pathophysiological process [4]. Unlike other powerfully regenerative organs, such as the heart or liver, injured kidneys that suffer from AKI usually do not have the capacity to completely repair themselves [5]. Maladaptive kidneys will undergo fibroblast activation and the deposition of the extracellular matrix, which may contribute to a vicious cycle of fibrogenesis and nephron loss and ultimately induce the development of CKD [6, 7]. For this reason, it is easy to understand the fact that AKI patients who survive the acute phase will bear a 13-fold higher risk of developing CKD in their lifetime. For those patients with AKI at the RIFLE failure stage, the risk of progression to CKD is up to 41 times higher [8]. The phenomenon of progression to CKD after AKI is termed the AKI-CKD transition [9].

During the last decade, multiple therapeutic strategies have been explored for targeting the AKI-CKD transition; however, few faithful pharmacologic agents have been proven to be able to effectively block this transition. Interventions with hypoxia-inducible factor (HIF), vascular endothelial growth factor (VEGF), or nuclear factor erythroid-2-related factor 2 (Nrf2) present promising outcomes in some studies, but contradictions still exist [10–13]. Currently, the strongest evidence for the management of disease progression still comes from the way in which we manage established CKD, namely, renin-angiotensin system inhibitor (RASI). However, this strategy provides no help for renal regeneration [14, 15]. Exploring novel interventions that have the capacity to promote renal regeneration and halt AKI-CKD transition is an urgent need.

MSCs are a type of stem cell which are characterized by their robust capacities of self-renewal, regeneration, proliferation, and three-lineage differentiation [16]. Multiple animal injury models, including the lung, liver, and Alport’s syndrome, have confirmed that MSCs are able to reverse organ fibrosis [17–19]. Compared with pharmacologic interventions, which target only one single aspect of the highly complex pathophysiological process during the AKI-CKD transition, MSCs may have the advantage of presenting multiple regenerative effects for organ protection [20]. It is thought that MSCs possess intrinsic properties of immunomodulation, proangiogenesis, anti-inflammation, anti-apoptosis, and anti-oxidation, which are greatly beneficial for limiting acute injury and reversing chronic progression [21–23].

However, to date, most studies in the field of regenerative medicine are still confined to only evaluating the therapeutic effects of MSCs on either AKI or CKD [24, 25]. Few research studies have paid attention to issues regarding the AKI-CKD transition. It seems that a knowledge gap still exists regarding the connection between AKI and CKD. Given that this concept was just proposed in the last decade, it is also comprehensible to understand the situation [26]. Fortunately, the phenomenon of chronic sequelae of AKI is receiving more and more attention in the last few years based on the fact that there has been a dramatic increase in research. Regenerative medicine physicians are also wondering how to regard the role of MSCs in this area. Due to these conditions, we collected associated evidence about the administration of MSCs in AKI-CKD transition. This is the first review article related to this issue thus far. By summarizing available articles and discussing the underlying mechanisms in this review, we intend to provide an integrated and up-to-date view of the application of MSCs in the AKI-CKD transition and call for a focus on issues related to long-term ramifications after AKI, with the aim of improving prognosis in AKI patients and delaying the progression to CKD.

## Maladaptive repair after AKI and the mechanisms involved in the transition

**Fig. 1:**
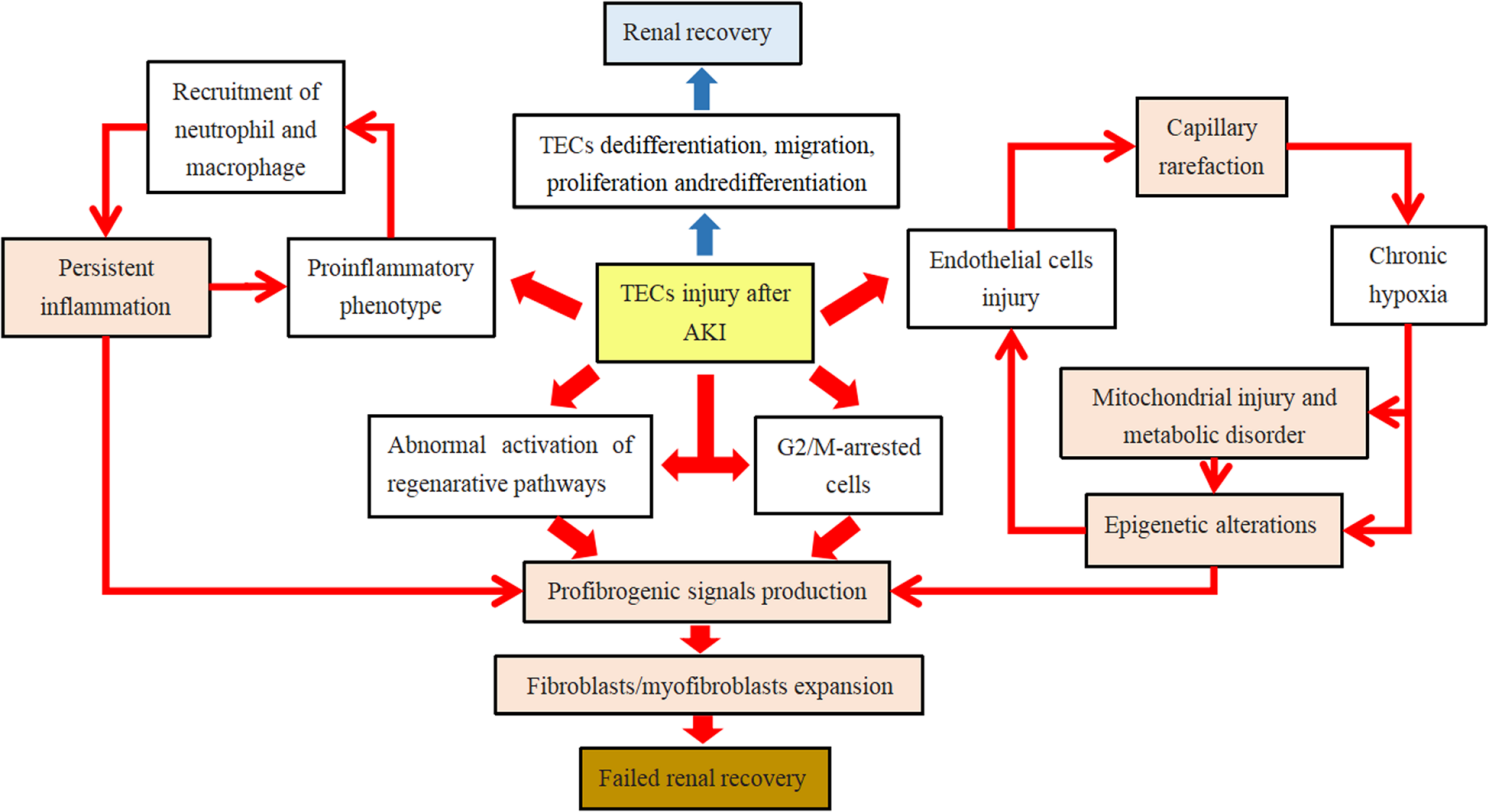
Adaptive and maladaptive response in TECs after AKI. Under certain circumstances, injured TECs are able to dedifferentiate, migrate, proliferate, and finally redifferentiate into normal TECs, promoting renal recovery (blue arrows). While AKI is severe or episodes are frequent, TECs may lose their regenerative capacity and undergo maladaptive repair. The major pathophysiological alterations during this process include capillary rarefaction, mitochondrial injury and metabolic disorder, epigenetic alterations, persistent inflammation, and other effects. These changes may interact with each other and can ultimately result in profibrogenic signal production, fibroblast/myofibroblast expansion, and failed renal recovery (red arrows)

Several years ago, it was thought that the repair process after AKI might be fully adaptive and that surviving patients who recovered renal function would not suffer from long-term consequences [27]. Lineage-tracing studies indicated that tubular epithelial cells (TECs) were thought to be the main source of cell regeneration after injury [28, 29]. During the repair process, surviving TECs underwent dedifferentiation, migration along the basement membrane, proliferation, and finally redifferentiation. After these orchestrated reparative processes, damaged TECs were replaced with functional new ones, and a refreshed steady state was established [30, 31]. However, due to the importance of TECs, a maladaptive response in TECs is also thought to occupy a central position in the transition from AKI to CKD, regardless of the diverse injuries in the blood vessels, glomeruli, and inflammatory/immune cells [26]. An episode of injury will lead to capillary rarefaction, mitochondrial injury and metabolic disorder, epigenetic alterations, persistent inflammation, profibrogenic signal production, and fibroblast/myofibroblast expansion, which will ultimately cause the development of CKD (Fig. 1) [32–38]. In the following section, we would like to discuss these chronic sequelae after AKI and the relevant biochemical pathways related to its progression.

### Capillary rarefaction

Unlike TECs, vascular endothelial cells possess less regenerative potential [39]. Accompanied by low expression levels of VEGF after AKI, one AKI strike may result in disastrous consequences to the renal tissue microvasculature [38, 40]. It was reported that a massive reduction of capillary density occurred after inducing AKI in a rat model [41]. Capillary rarefaction could exacerbate tissue hypoxia, both of which no doubt play critical roles in promoting interstitial fibrosis and the progression to CKD [42, 43]. Based on these facts, Kapitsinou et al. applied pharmacologic HIF agonists to prevent the development of fibrosis after AKI and showed positive results, which in turn verified this hypothesis [10].

### Mitochondrial injury and metabolic disorder

Capillary rarefaction and hypoxia initiate mitochondrial injury and metabolic disorder, especially in proximal TECs and medullary thick ascending limb TECs, which largely rely on oxidative metabolism to provide the vast amount of adenosine triphosphate (ATP) they require for tubular reabsorption [44]. Pathologically, injured mitochondria present with reduced numbers, fragmented shape, and formation of autophagosomes [45, 46]. Functionally, cells switched from oxidative phosphorylation to glycolysis, inducing fat accumulation, together with the downregulation of multiple electron transport chain and ATP synthesis genes [45, 47]. Moreover, the release of dangerous molecules by mitochondria, such as reactive oxygen species (ROS), would be activated if long-term injury existed, which sustained chronic inflammation and delayed renal recovery [48, 49]. All of these alternations in mitochondria contribute to mediating glomerulosclerosis and interstitial fibrosis after AKI.

### Epigenetic alterations

In addition to the metabolic and structural changes in the mitochondria, disturbances in epigenetic modifications can also be activated after AKI. Considering that histone deacetylase is a key regulator of the expression of fibrosis-related genes, Novitskaya et al. and Cianciolo et al. tried to determine if aberrant histone deacetylase contributed to AKI-CKD transition in two different AKI-CKD transition models. Acceleration of AKI recovery and prevention of post-injury fibrosis were observed after the inhibition of histone deacetylase [50, 51]. Apart from histone deacetylase, there is an extensive crosstalk between histone modification and DNA methylation [52]. A subsequent study by Tampe et al. showed that the administration of low-dose hydralazine efficiently catalyzed RASAL1 demethylation and inhibited tubulointerstitial fibrosis [53]. The results from these studies confirmed the crucial roles that epigenetic alterations played in AKI-CKD progression.

### Persistent inflammation

AKI is no doubt closely linked with inflammation. Tubulointerstitial inflammation can be observed in almost all types of AKI in the early stage, whereas persistent inflammation inevitably results in the progression of fibrosis. The interaction between tubulointerstitial inflammation and TECs is a key determinant in the development of persistent inflammation. Multiple identical pattern recognition receptors, including Toll-like receptors (TLRs), Nod-like receptors, and NLRP3 inflammasomes, were found to be widely expressed in TECs [54]. During the early stage of AKI, tubulointerstitial inflammation triggered by pathologic factors, such as ischemia, toxins, or proteinuria, could activate the TLR2-MyD88-NF-kB pathway and upregulate NLRP3 inflammasomes [55, 56]. These alternations in TECs transformed them into a proinflammatory phenotype and elicited the secretion of various proinflammatory mediators, including tumor necrosis factor (TNF)-α, IL-6, IL-1β, IL-15, IL-16, and VEGF, among others [57–61]. The release of proinflammatory cytokines recruited neutrophils and infiltrating macrophages, polarized macrophages/dendritic cells, facilitated the immune response, and led to persistent AKI and subsequent CKD [62, 63]. These results support a role for persistent inflammation in the pathogenesis of the AKI-CKD transition.

### Profibrogenic signal production

Multiple signal pathways can be activated during the recovery phase of AKI due to the requirement of cell regeneration, but all of these pathways should be ceased following renal recovery. Abnormal activation of these pathways can induce pathological outcomes. Examples include Wnt/b-catenin and miR-21/phosphatase and tensin homolog (PTEN) signaling. The activation of Wnt-4/b-catenin in the acute phase of injury allows injured cells to re-enter the cell cycle and regulates cell proliferation, which is helpful in renal recovery [64, 65]. However, sustained activation of Wnt signaling is accompanied by the development of interstitial myofibroblast activation and excessive extracellular matrix deposition [66]. A similar situation was also observed in the case of miR-21/PTEN signaling [67, 68]. Disturbance in the regulation of these pathways transformed regenerative signals into profibrogenic signals.

Apart from the abovementioned mechanism, the expansion of profibrogenic signals can also be driven by G2/M-arrested cells. Unlike adaptive repair, which induced surviving TECs to undergo continuous four-phase mitosis for proliferation (G0, G1, S, G2 and M), particularly stressful conditions during AKI may lead some TECs to remain arrested in the G2/M phase. These TECs are active in the production of profibrogenic signals, such as transforming growth factor-β (TGF-β), c-jun NH2-terminal kinase (JNK), and epidermal growth factor receptor (EGFR), which provide a pathophysiological link between AKI and CKD [35, 69, 70].

### Fibroblast/myofibroblast expansion

Fibroblast/myofibroblast expansion is the final procedure for the development of fibrosis. After stimulation by the abovementioned pathophysiological alternations, including capillary rarefaction, mitochondrial injury and metabolic disorder, epigenetic alterations, persistent inflammation, and profibrogenic factor production, interstitial precursor cells finally differentiate into fibroblasts/myofibroblasts. Although the origin of fibroblasts/myofibroblasts is still under debate, pericytes seem to be the major source. Normally, pericytes are cells of mesenchymal origin with branching processes that form junctions between the capillaries and tubules [71]. Under stressful conditions such as AKI, harmful signals, such as platelet-derived growth factor receptor-β (PDGFR-β), VEGF receptor 2, or α-engagement, can drive pericytes to detach from the capillaries. Detached pericytes spread, migrate, and transform into myofibroblasts [32, 72]. In addition to pericytes, some research studies have also regarded TECs, endothelial cells, or bone marrow cells as other sources of fibroblasts/myofibroblasts [7, 38]. The continuous expansion of fibroblasts/myofibroblasts, with excessive production of collagen, widens the normal tubulointerstitial space and injured endothelium regresses, causing kidney fibrosis [73].

## MSCs are a promising candidate for the management of the AKI-CKD transition

Above, we discussed the pathophysiological alterations during the AKI-CKD transition. It should be noted that the abovementioned pathophysiological changes are not merely separated processes; rather, they interact with each other in a complicated network. For example, as described by Liu et al. in their article, TECs under injury can undergo a proinflammatory phenotype change and drive interstitial inflammation. In turn, activated infiltrating immune cells can also induce TEC necrosis and exaggerated renal injury [74]. Similarly, Venkatachalam et al. also demonstrated that although renal epithelial dysfunction with ensuing paracrine activity is the initial issue in this scheme, subsequent interactions between different pathophysiological processes, such as inflammation, fibroblasts, and capillary rarefaction, can form a self-reinforcing cycle feedback [75]. These lines of evidence make it difficult to assign their roles as “chicken and egg” in many cases. Additionally, for this reason, it is not hard to understand the limited therapeutic effects of drug interventions in the AKI-CKD transition because one single drug approach commonly only targets one single pathway.

The advantage of MSC-based therapy over drug interventions in attenuating the AKI-CKD transition is due to its multipotent regenerative properties both in vitro and in vivo, which cover most of its highly complex pathophysiological processes. Although only scarce evidence has demonstrated that MSCs can replace injured tissues in vivo, the capacity to differentiate into all mesodermal lineage cells was well verified in vitro, and this potential was full of interest and imagination [76]. The most widely accepted proregenerative capability of MSCs relied on its paracrine/endocrine capacity. Accumulating evidence has emphasized that MSCs are active in the release of a number of cytokines/growth factors, such as hepatocyte growth factor (HGF), fibroblast growth factor (FGF), VEGF, TGF-β, and insulin-like growth factor type 1 (IGF-1) [77]. These biological molecules can exert anti-apoptotic, anti-oxidative, and pro-angiogenic effects, promoting local cell recovery [78–80]. Recent findings have also suggested that MSCs can alleviate renal injury by secreting extracellular vesicles (EVs), which is regarded as another special form of paracrine/endocrine response. EVs are important cell-to-cell interaction mediators and are rich in a broad variety of biologically active molecules, including lipids, proteins, and nucleic acids (e.g., mRNAs, miRNAs, and lncRNAs) [81]. After internalization, the biologically active molecules inside EVs can be transmitted into the target cells, changing their phenotype and exerting biological effects [82]. Except for the secretion of bioactive substances, MSCs can also act as an ideal vehicle for organelle delivery. For example, by transferring healthy mitochondria, defective native ones in injured renal cells can be substituted, and aerobic respiration can be revitalized [44]. Based on these regenerative properties, it is not surprising that MSCs are regarded as a promising candidate in the AKI-CKD transition. In the following section, we will talk about the application of MSCs in different AKI-CKD transition models and the mechanisms underlying their beneficial effects (Table 1). Most of the currently available data has come from ischemia-reperfusion (I/R) injury-induced AKI-CKD transition models.

**Table 1:** Evidence suggesting the beneficial effects of MSCs on the AKI-CKD transition

References	Year	Animal	Model	Renal outcomes
Zhu et al. [84]	2017	Mice	Unilateral I/R injury	↓Fibrosis; ↑kidney weight and size; ↓α-SMA and collagen-I; ↓TGF-β1/Smad3
Masoud et al. [85]	2012	Rats	Unilateral I/R injury	↓Fibrosis
Semedo et al. [86]	2010	Mice	Unilateral I/R injury	↓Fibrosis; ↓proteinuria; ↑renal areas; ↓a-SMA, vimentin, and FSP-1
Gatti et al. [88]	2011	Rats	Unilateral I/R injury with contralateral nephrectomy	↑Renal function; ↓fibrosis; ↓proteinuria
Du et al. [90]	2012	Rats	Unilateral I/R injury with contralateral nephrectomy	↑Renal function; ↓fibrosis
Zou et al. [89]	2014	Rats	Unilateral I/R injury with contralateral nephrectomy	↑Renal function; ↓fibrosis; ↓α-SMA
Alfarano et al. [91]	2012	Rats	Unilateral I/R injury with contralateral nephrectomy	↑Renal function; ↓fibrosis and tubular dilation; ↓α-SMA and MMP2
Zhou et al. [93]	2016	Rats	Unilateral I/R injury with contralateral nephrectomy	↑Renal function; ↓fibrosis; ↓α-SMA; ↓microvascular rarefaction
Tögel et al. [94]	2009	Rats	Bilateral I/R injury	↑Renal function; ↓fibrosis; ↓PAI-1 and TGF-β
Du et al. [96]	2013	Rats	Bilateral I/R injury	↓Fibrosis
Rodrigues et al. [97]	2017	Rats	Bilateral I/R injury	↑Renal function; ↓FENa; ↓urinary concentrating defect; ↓renal damage score; ↓senescence

*I/R* ischemia/reperfusion, *α-SMA* α-smooth muscle actin, *MMP2* matrix metalloproteinase 2, *FSP-1* fibroblast specific protein-1, *PAI-1* plasminogen activator inhibitor-1

### Application of MSCs in the unilateral I/R injury-induced AKI-CKD transition model

The unilateral I/R injury model is a widely used model in the study of the AKI-CKD transition. This model is induced by clamping the pedicle of only one kidney. Fibrosis of the renal tissues followed by the ischemic strike can be observed in this model, making it a reliable strategy to study the AKI-CKD transition [83].

Zhu et al. transferred adipose-derived MSCs (A-MSCs) into a mouse model of unilateral I/R injury-induced AKI-CKD transition. The administration of A-MSCs significantly alleviated the fibrosis and atrophy of renal tissues 28 days after the severe event of AKI. Molecularly, α-smooth muscle actin (α-SMA) and collagen-I were reduced at both the mRNA and protein levels, suggesting the attenuation of fibrosis. To further explore the mechanism underlying the beneficial effects of A-MSCs on the AKI-CKD transition, they assessed the expression of TGF-β, an important pro-fibrotic factor in renal tissues. As expected, a reduced expression of TGF-β1 and a decreased level of phosphorylation Smad3 were observed [84]. Similar outcomes were obtained by Masoud et al. In their study, animals injected with MSCs presented improved renal fibrosis after clamping the left renal pedicle for 45 min [85]. MSCs are the main component of bone marrow mononuclear cells (BMMCs). BMMC therapy after unilateral I/R injury could significantly halt its progression to chronic fibrosis. After 6 weeks of transplantation, although the serum creatinine level was not changed, fewer fibrosis areas and less proteinuria together with larger renal areas in the injured kidneys were observed in animals that received BMMC therapy when compared with untreated animals. At the molecular level, pro-fibrotic molecules, such as α-SMA, vimentin, and fibroblast-specific protein-1 (FSP-1), presented similar patterns in the kidneys of BMMC-treated animals, suggesting a protective role of BMMCs in attenuating the AKI-CKD transition [86].

### Application of MSCs in the unilateral I/R injury with contralateral nephrectomy injury-induced AKI-CKD transition model

All of the abovementioned studies lack an outcome in renal function. This is due to a major disadvantage of the unilateral I/R injury-induced AKI-CKD transition model. With a functional and compensatory contralateral kidney, it is always difficult to monitor the renal functional decline. One way to partially overcome this problem is to remove the contralateral kidney on the basis of unilateral I/R injury, namely, unilateral I/R injury with contralateral nephrectomy injury [87].

The first study evaluating the long-term effects of MSC therapy on the AKI-CKD transition in this model was conducted by Gatti et al. in 2011. The administration of microvesicles (MVs) derived from MSCs immediately after right nephrectomy with left renal pedicle clamping significantly mitigated long-term kidney injury 6 months later. Proteinuria and fibrotic renal tissues in MV-treated rats were also both significantly lower than in the control group [88]. Similarly, the results originated from studies by Zou et al. and Du et al. indicated that a single injection of Wharton’s jelly-derived MSCs (WJ-MSCs) after unilateral I/R injury with contralateral nephrectomy could reverse renal fibrosis and protect against CKD [89, 90]. To closely examine the protective mechanism of MSC therapy, Alfarano et al. evaluated the development of CKD in their model of the AKI-CKD transition. Transplantation with bone marrow-derived MSCs (BM-MSCs) improved renal function and modified renal remodeling in parallel with decreased accumulation of α-SMA and metalloproteinase 2 (MMP2). Additionally, the authors further demonstrated that these beneficial mechanisms of MSC therapy might not account for its immunomodulatory or anti-inflammatory properties because of the usage of the immunosuppressive drug cyclosporine in their study, which indicated a direct activity of MSCs in the inhibition of fibrosis in renal tubular cells [91]. The stromal vascular fraction (SVF) was considered to be a rich source of MSC and also presented promising potential in delaying the AKI-CKD transition [92]. Zhou et al. attempted to engraft SVF into the kidneys of rats suffering from unilateral I/R injury with contralateral nephrectomy. Six months later, declines in renal function and progression of fibrosis were both delayed by the administration of SVF, which strongly inhibited microvascular rarefaction [93].

### Application of MSCs in the bilateral I/R injury-induced AKI-CKD transition model

Compared with the unilateral I/R injury model and the unilateral I/R injury with contralateral nephrectomy model, the bilateral I/R injury model is a more accurate model to study the AKI-CKD transition in humans. The bilateral I/R injury model is induced by the block of renal vessels in both kidneys, which may make the renal hemodynamic changes more relevant to human pathophysiological situations. Similar with the results obtained in the unilateral I/R injury with contralateral nephrectomy model, improvement of renal function and abrogation of renal fibrosis were observed in rats that received MSCs after suffering from bilateral I/R injury. Profibrotic genes, such as TGF-β and tissue inhibitor of matrix metalloprotease-1 (TIMP-1), were also expressed in these kidneys at significantly lower levels, suggesting the anti-fibrotic effects of MSCs. However, after the effective knockdown of VEGF by siRNA in engrafted MSCs, all of the beneficial effects disappeared, indicating the importance of the VEGF pathway against a maladaptive repair in renal I/R injury [94]. In addition to VEGF, HGF was another key factor in mediating the protective effects of MSCs against the AKI-CKD transition. A previous study reported that HGF could efficiently block the TGF-β1/Smad signaling [95]. To verify this hypothesis, a rat model of unilateral I/R injury was established. After 2 days, a single injection of WJ-MSCs was performed, and the ratio of HGF/TGF-β1 was measured. Six weeks later, although without a change in the serum creatinine value, cell treatment significantly mitigated renal fibrosis triggered by the unilateral I/R injury. In terms of mechanism, an upregulated ratio of HGF/TGF-β1 was unambiguously observed after MSC transplantation. Via these experiments, the authors confirmed that MSC therapy might exert an important effect on the balance between HGF and TGF-β1 during fibrogenesis [96]. Renal aging and Klotho are important factors in mediating the development of CKD. To evaluate their role in mediating the therapeutic effects of MSCs during the AKI repair process, Rodrigues et al. intraperitoneally injected MSCs into unilateral I/R injury-induced AKI rats. At day 49, senescence-related proteins and Klotho expression were both ameliorated in rats submitted to MSC therapy, which further helped to reverse renal dysfunction, urinary concentrating defect, and renal damage [97].

## Attempts with MSC therapy in clinical settings and challenges met in translation

Above, we discussed the success of MSC therapy in preclinical models. What about the role of MSCs in clinical settings? Unfortunately, at this point, a clinical study assessing the role of MSC-based therapy in the field of the AKI-CKD transition is still lacking. However, as we know, maladaptive repair after AKI is a major risk factor for the development of the AKI-CKD transition. Here, we list the currently available clinical trials using MSC applications in AKI (Table 2). NCT00733876 was the first clinical trial assessing the safety and therapeutic effects of MSC transplantation in AKI patients. Allogeneic MSCs were prophylactically injected into 16 patients who were at high risk of developing AKI after undergoing on-pump cardiac surgery [98]. The administration was safe, and the outcome was positive, which encouraged the team to conduct a larger number, phase II, randomized, double-blind, multicenter trial in 2017 (NCT01602328) [99]. This trial included a total of 156 adult subjects who developed AKI after cardiac surgery. However, MSC treatment did not aid in renal recovery, and AKI duration, dialysis prevalence, and 30-day all-cause mortality were comparable between the two groups. A highly anticipated study (NCT01275612) aimed at evaluating the feasibility and safety of the use of MSCs in the treatment of cisplatin-induced AKI was withdrawn recently. Based on these contradictory results, a third ongoing trial (NCT03015623) is currently recruiting. More research is needed to answer the question of whether MSC-based therapy can be effective in reducing the AKI-CKD transition in humans.

**Table 2:** Available clinical trials of the application of MSCs in AKI

ClinicalTrials.gov identifier	Year	Aim	MSC type	Enrollment	Phase	Status	Outcomes
NCT00733876	2008	For the prevention of developing AKI after cardiac surgery	Allogeneic	16	Phase I	Completed	Safe and effective
NCT01602328	2017	For the treatment of AKI after cardiac surgery	Allogeneic	156	Phase II	Terminated	Safe but not effective
NCT01275612	2018	For the treatment of cisplatin-induced AKI in cancer patients	Allogeneic	NM	Phase I	Withdrawn	NM
NCT03015623	2018	For the treatment of AKI regardless of the underlying reasons	Allogeneic MSCs combined with a biologic device	24	Phase I	Recruiting	Date not published

*MSCs* mesenchymal stem cells, *AKI* acute kidney injury, *NM* not mentioned

Why are promising preclinical results rarely verified in clinical settings? In our opinion, the restricted cell function after transplantation is a major factor in the poor translation outcomes. As we discussed in a previous article, the main reasons for the limited clinical efficacy were the low engraftment, the poor survival rate, and the impaired paracrine capacity of injected cells in vivo [100]. The majority of grafted MSCs might be trapped in the lungs, liver, and spleen. For those cells engrafted into the target tissues, the harsh microenvironment in vivo due to the activation of anoikis, ischemia, inflammation, and ROS production still would induce apoptosis in over 90% of transplanted MSCs in 1 week [101]. Impaired MSC potency/biological activity is also common in vivo. Different preconditioning strategies have been designed to increase MSC engraftment, survival, and paracrine capacity in vivo and have presented wonderful results [102]. These facts have provided an explanation for the poor therapeutic effects in clinical trials.

In addition to the abovementioned difficulties during clinical translation, some failures were also met in animal models (Table 3). In a feline model of unilateral I/R injury-induced AKI, Rosselli et al. demonstrated that renal function, urine protein creatinine ratio (UP/C), SMA, and histopathologic scoring were all comparable in cats receiving MSC treatment and the control group [103]. Similarly, the results from a study by Fang et al. also indicated that hematopoietic lineage marrow cells (HLMCs) but not MSCs appeared to influence the course of HgCl_2_-induced AKI in mice [104]. Along with these negative results, the adverse effects deserve more attention. The renin-angiotensin system (RAS) has been reported to be involved in several key steps of bone marrow cell maturation processes [105]. To evaluate whether pathological RAS activation will have an impact on the therapeutic functionality of BM-MSCs, Kankuri et al. transplanted BM-MSCs derived from rats with increased RAS activation into rats suffering from bilateral I/R injury-induced AKI. Unexpectedly, a dramatic deterioration of renal function was observed in the treatment group. Alterations in the host’s RAS system induced a pro-inflammatory phenotype in BM-MSCs, which might compromise their therapeutic effects [106]. Meanwhile, although not conducted in an AKI setting, Kim et al. reported a case of a rapid deterioration of renal function after MSC treatment [107]. These lines of evidence highlighted the ineffectiveness and potential nephrotoxicity of MSC-based therapy; thus, it should be examined more closely before its translation into clinical applications.

**Table 3:** Failed attempts of MSC treatment in animal models

Reference	Year	Sample	Model	Renal outcomes
Rosselli et al. [103]	2015	Cats	Unilateral I/R injury	No changes in renal function, UP/C, SMA, and histopathologic scoring
Fang et al. [104]	2008	Mice	HgCl_2_	No contribution to renal tubular cells regeneration
Kankuri et al. [106]	2015	Rats	Bilateral I/R injury	Exacerbation of renal function

*MSCs* mesenchymal stem cells, *I/R* ischemia-reperfusion, *UP/C* urine protein: creatinine, *SMA* smooth muscle actin

Based on the lines of evidence mentioned above, there is still a long way to go before considering MSCs as a realistic clinical tool for the AKI-CKD transition. The challenges that need to be addressed here may also be of relevance for other approaches that utilize cell-based strategies in many other cases. First, a standard MSC regimen is needed. What kind of MSCs will serve as the best tissue source? BM-MSCs, umbilical cord-derived MSCs (UC-MSCs), A-MSCs, or induced pluripotent stem cell-derived MSCs (iPSC-MSCs)? Autologous or allogeneic? What is the best delivery route for transplantation? Intravenous, intra-arterial, intraperitoneal, or intrarenal injection? What is the best dosage and timing for AKI-CKD transition therapy? These discordances may induce apparent discrepancies in reports in the literature. Second, rigorous criteria for cell extraction, storage, and characterization are still lacking; as a result, MSCs are highly heterogeneous cells. Last but not least, safety expectations, including tumorigenicity and immunological compatibility, should be considered. Although there has not yet been a report of de novo tumor formation or significant immunogenicity in vivo following MSC injection in humans, these issues should always be kept in mind. Additional studies are warranted to address these issues.

## Conclusion and future perspectives

By summarizing the currently available research, we would like to conclude that therapeutic strategies based on MSCs are no doubt promising for the management of the AKI-CKD transition. However, before the clinical application of MSCs, some issues remain to be solved in future research studies.

First, although most studies support the beneficial role of MSCs in the management of the AKI-CKD transition, the heterogeneity of cell populations makes MSC transplantation a fundamentally different strategy from most pharmacologic interventions. Different MSC subtype characteristics, differences in protocols used to obtain the cells, the lack of a standardized MSC preparation method, and other inconsistencies might help us to explain why there are still some contradictory results in the literature. The complexity of different therapeutic regimens could also contribute to the controversial results. Conducting coordinated studies comparing the effects of distinct subpopulations of MSCs in therapeutic therapy regimens may give us a clearer insight into these questions.

**Fig. 2:**
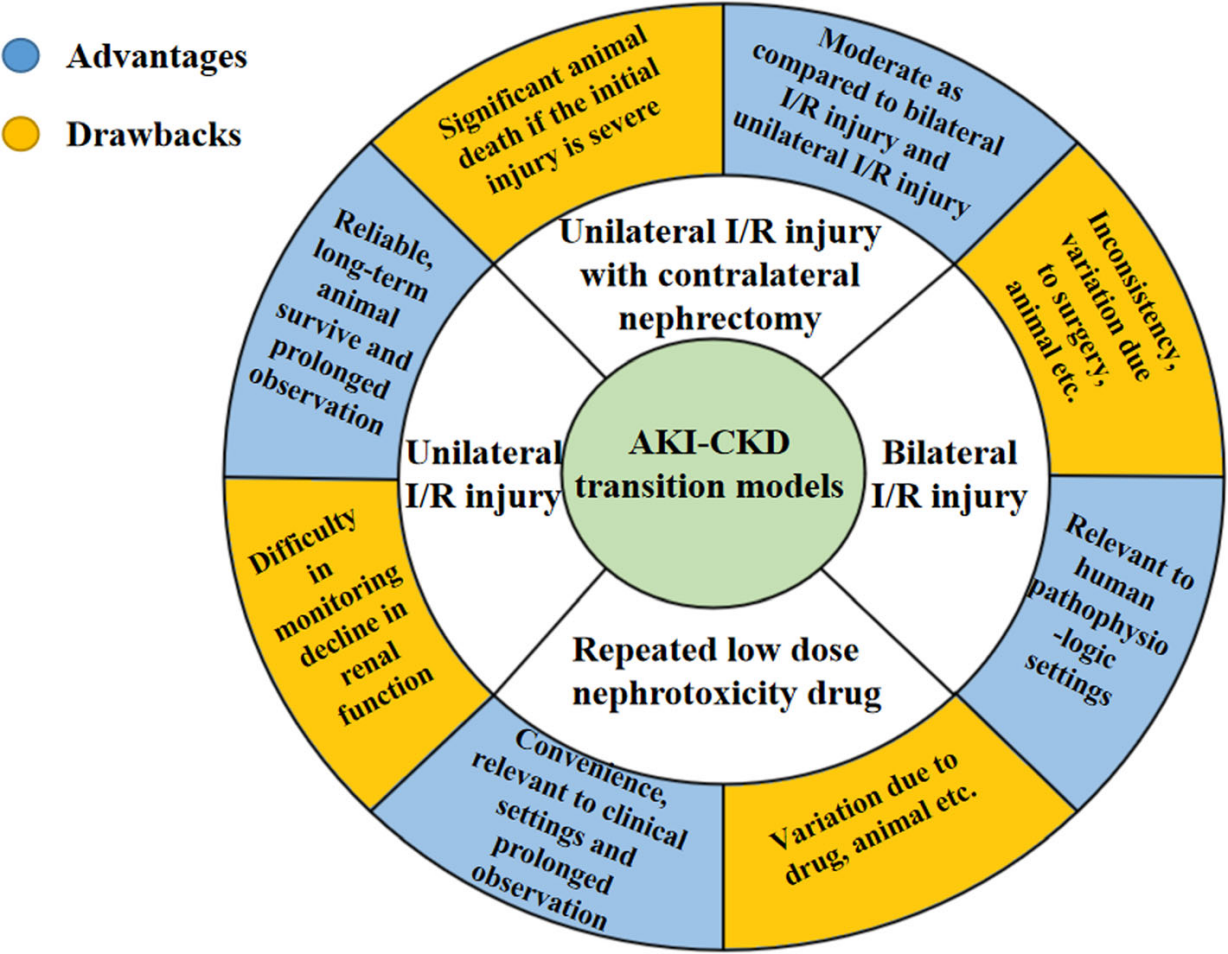
Advantages and pitfalls of different animal models in AKI-CKD transition research

The second issue regards the model of the AKI-CKD transition. I/R injury models are still the best developed and most widely applied animal models in the research field of the AKI-CKD transition. According to different surgical procedures, these models can be classified into bilateral I/R injury, unilateral I/R injury, and unilateral I/R injury with contralateral nephrectomy. In addition to I/R injury models, repeated low-dose nephrotoxicity drug stimulation may also cause long-term side effects in the kidneys and lead to an AKI-CKD transition. The advantages and pitfalls of these models were mentioned above and are summarized in Fig. 2. While I/R injury models provide the consistency, reliability, and capacity of long-term observation, nephrotoxic models are also a good supplement to I/R injury models for understanding the different causes and mechanisms of the AKI-CKD transition, especially in cancer patients who intermittently receive cisplatin therapy.

Another important issue is the definition of the AKI-CKD transition. A consensus about the definition of AKI recovery is still lacking. Serum creatinine and estimated glomerular filtration rate (eGFR) are used as traditional biomarkers for the judgment of the existence of renal injury. However, due to the low sensitivity of serum creatinine and eGFR in diagnosing kidney disease, these markers are insufficient for precisely reflecting the pathophysiological processes during the AKI-CKD transition. To overcome this shortcoming, the 13th Acute Dialysis Quality Initiative Meeting described adaptive repair as a phenomenon without long-term sequelae in renal function and structure over a period of three months after AKI [9]. However, this definition largely depends on renal biopsy, which may limit its clinical application due to its invasive property. In our opinion, establishment of a standard definition for the AKI-CKD transition by application of other novel biomarkers, such as neutrophil gelatinase-associated lipocalin (NGAL), kidney injury molecule-1 (KIM-1), nephrin, podocalyxin, IL-18, and other biomarkers, could help to provide early diagnosis and allow for a timely intervention for these patients, which is urgently needed.

Finally, we need to establish a post-AKI monitoring system. Despite developing in the field of pharmaceutical research, the best way to reduce AKI-CKD continuum is still prevention. However, given that the severity, duration, and frequency of repeat episodes of AKI are critical points of its poor outcome, the clinical follow-up rate of AKI survivors is still extremely low at present [108, 109]. While advancing potential therapies, in the meantime, there is an urgent need to define those patients who are most at risk of developing CKD after an episode of AKI. Exploring novel protocols, including essential components and precise timing for monitoring patients who suffer from AKI, is of great importance.

In conclusion, although there is still no reliable therapeutic approach for the treatment of the AKI-CKD transition at present, MSCs have the potential to block AKI-CKD transition, at least partially. Randomized controlled clinical trials should be initiated, and we call for more research in this area.

## Data Availability

Not applicable

## Abbreviations

AKI: Acute kidney injury
A-MSCs: Adipose-derived MSCs
ATP: Adenosine triphosphate
BMMCs: Bone marrow mononuclear cells
BM-MSCs: Bone marrow-derived MSCs
CKD: Chronic kidney disease
EGFR: Epidermal growth factor receptor
eGFR: Estimated glomerular filtration rate
EVs: Extracellular vesicles
FGF: Fibroblast growth factor
FSP-1: Fibroblast-specific protein-1
HGF: Hepatocyte growth factor
HIF: Hypoxia-inducible factor
HLMCs: Hematopoietic lineage marrow cells
I/R: Ischemia-reperfusion
IGF-1: Insulin-like growth factor type 1
iPSC-MSCs: Induced pluripotent stem cell-derived MSCs
JNK: c-jun NH2-terminal kinase
KIM-1: Kidney injury molecule-1
MMP2: Metalloproteinase 2
MSCs: Mesenchymal stem cells
MVs: Microvesicles
NGAL: Neutrophil gelatinase-associated lipocalin
Nrf2: Nuclear factor erythroid-2-related factor 2
PAI-1: Plasminogen activator inhibitor-1
PDGFR-β: Platelet-derived growth factor receptor-β
PTEN: Phosphatase and tensin homolog
RAS: Renin-angiotensin system
RASI: Renin-angiotensin system inhibitor
ROS: Reactive oxygen species
SVF: Stromal vascular fraction
TECs: Tubular epithelial cells
TGF-β: Transforming growth factor-β
TIMP-1: Tissue inhibitor of matrix metalloprotease-1
TLRs: Toll-like receptors
TNF-α: Tumor necrosis factor-α
UC-MSCs: Umbilical cord-derived MSCs
UP/C: Urine protein creatinine ratio
VEGF: Vascular endothelial growth factor
WJ-MSCs: Wharton’s jelly-derived MSCs
α-SMA: α-Smooth muscle actin
